# Supplementary material for: The mechanism of peptidoglycan O-acetylation in Gram-negative bacteria typifies bacterial MBOAT-SGNH acyltransferases[image]
Source: J Biol Chem. 2025 Apr 23;301(6):108531. doi: 10.1016/j.jbc.2025.108531 (PMC12148441; doi:10.1016/j.jbc.2025.108531)
Supplement: Supporting information [file mmc1.pdf]

Supporting Information for:

The mechanism of peptidoglycan *O*-acetylation in Gram-negative bacteria typifies bacterial MBOAT-SGNH acyltransferases

Alexander C Anderson[a,1], Bailey J. Schultz[b†], Eric D. Snow[b†], Ashley S Brott[a], Stefen Stangherlin,[a,2], Tyler Malloch[c], Jalen R. London[b], Suzanne Walker[b], Anthony J Clarke[a,c]

[a]Department of Molecular and Cellular Biology, University of Guelph, Guelph, Ontario Canada N1G 2W1

[b]Department of Microbiology, Blavatnik Institute, Harvard Medical School, Boston, MA, USA

[c]Department of Chemistry & Biochemistry, Wilfrid Laurier University, Waterloo, Ontario Canada N2L 3C5

[1]Present address, Michael DeGroote Institute for Infectious Disease Research, McMaster University, Hamilton, ON L8S 4K1, Canada

Department of Biochemistry and Biomedical Sciences, McMaster University, Hamilton, ON L8S 4K1, Canada

[2]Present address, Department of Chemistry, University of Waterloo, Waterloo, Ontario Canada N2L 3G1

[†]These authors contributed equally

Corresponding author: Anthony J. Clarke
Email: ajclarke@wlu.ca

This file includes:

Tables S1 and S2
Figures S1 to S15
SI References

Table S1. X-ray data collection and refinement statistics.

Dataset	*Ng*PatB$_{\Delta100}$ SeMet	*Ng*PatB$_{\Delta100}$ Native	*Ng*PatB$_{\Delta100}$ MeS	*Cj*PatB$_{\Delta113}$ Native
**Data Collection**				
Beamline	CLS 08ID-1	CLS 08ID-1	CLS 08ID-1	CLS 08ID-1
Wavelength (Å)	0.97828	0.97949	0.97828	0.97949
Space group	$P\ 3_2$	$P\ 3_2$	$P\ 3_2$	$P\ 6_1$
Unit cell parameters (Å/°)	$a = b$ = 144.5<br>c = 77.95<br>$\alpha = \beta$ = 90<br>γ = 120	$a = b$ = 144.9<br>c = 80.14<br>$\alpha = \beta$ = 90<br>γ = 120	$a = b$ = 145.7<br>c = 78.41<br>$\alpha = \beta$ = 90<br>γ = 120	$a = b$ = 55.83<br>c = 136.44<br>$\alpha = \beta$ = 90<br>γ = 120
Resolution range (last shell) (Å)	49.15 – 1.5 (1.55 – 1.50)	49.15 – 1.30 (1.36 – 1.19)	49.15 – 1.80 (1.86 – 1.80)	48.35 – 1.90 (1.968 – 1.90)
Total number of reflections	307956	829759	278624	386275
Number of unique reflections	50486	78745	29553	18944
Redundancy	6.1	10.5	32	20.4
Completeness (last shell) (%)	99.03 (99.93)	99.96 (99.85)	99.99 (99.93)	99.98 (100.00)
Average I/σ (I) (last shell)	7.1 (3.0)	17.1 (6.6)	10.1 (2.60)	28.39 (9.23)
R_{merge} (last shell) (%)[1]	9.7 (81)	7.7 (88)	6.2 (66)	9.9 (71)
$CC_{1/2}$ (last shell)[2]	0.998 (0.0918)	0.999 (0.974)	0.996 (0.988)	0.999 (0.977)
**Refinement**				
Resolution range (Å)	49.21 – 1.50	41.83 – 1.30	49.15 – 1.8	39.45 – 1.9
R_{work}/R_{free} (%)[2]	20.4 / 22.2	16.6 / 18.9	19.6 / 22.9	16.8 / 20.4
Number of atoms	1909	1991	1890	1857
Protein	1758	1782	1754	1753
Water	151	204	132	103
Ligand	0	5	4	1
Average B-factor (Å^2)[3]	18.9	24.5	26.2	21.7
Protein	18.3	23.6	25.6	24.1
Water	25.7	32.3	32.7	30.4
Ligand	N/A	47.8	37.7	30.0
RMS Bond lengths (Å)	0.006	0.005	0.006	0.007
RMS Bond angles (°)	0.805	0.764	0.711	0.770
Ramachandran favoured (%)	97.21	97.27	97.21	97.17
Ramachandran allowed (%)	2.79	2.73	2.79	2.83
PDB accession ID	7TLV	7TJB	7TRR	8TLB

Values in parentheses correspond to the highest resolution shell.

I/σ(I): intensity of a group of reflections divided by the standard deviation of those reflections.

[1]$R_{merge} = \sum\sum |I(k) - \langle I \rangle| / \sum I(k)$, where I(k) and $\langle I \rangle$ represent the diffraction intensity values of the individual measurements and the corresponding mean values. The summation is over all unique measurements.

[2]$R_{work} = \sum ||F_{obs}| - k|F_{calc}|| / |F_{obs}|$, where F_{obs} and F_{calc} are the observed and calculated structure factors, respectively. R_{free} is the sum extended over a subset of reflections excluded from all stages of the refinement.

[3]As calculated using MolProbity (1).

Table S2: Strains, plasmids, and primers used in this study.

Strains	Description or characteristics	Source or reference
C. jejuni 81-176	Wild type *C. jejuni* strain isolated from a diarrheic patient	2
E. coli DH5α	Laboratory strain used for cloning: F– φ80*lac* ZΔM15 Δ(*lac* ZYA-*arg* F)U169 *rec* A1 *end*A1 *hsd*R17(rK–, mK+) *pho* A *sup* E44 λ–thi-1 *gyr*A96 *rel* A1	Thermo Fisher
E. coli C43 (DE3)	Laboratory strain used for overexpression: F–*omp* T *hsd*SB (rB–, mB–) *gal dcm* (DE3), contains further mutations for overexpression of toxic proteins	3
E. coli BL21 (DE3)	Laboratory strain used for overexpression: F–*omp* T *hsd*SB (rB–, mB–) *gal dcm rne* 131 (DE3)	Invitrogen
E. coli Stellar	*F- endA1 supE44 thi-1 recA1 relA1 gyrA96 phoA Φ80dlacZΔM15 Δ(lacZYA-argF) U169* Δ(*mrr-hsdRMS-mcrBC*) *ΔmcrA λ-*	Takara Bio
N. gonorrhoeae FA1090	Serum-resistant, proline-requiring strain isolated from a patient with probable disseminated gonococcal infection	4
**Plasmids**		
pBAD	T7-promoter driven *E. coli* vector with *araBAD* promoter for N-terminal His_6-fusion proteins	Invitrogen
pET28a(+)	T7-promoter driven *E. coli* expression vector for His_6-fusion proteins	Novagen
pET26b(+)	T7-promoter driven *E. coli* expression vector with *pelB* signal sequence for periplasmic localization of His_6-fusion proteins	Novagen
pACAB7	pBAD HisA expression plasmid containing His_6-SUMO tagged *Ng*Pat$B_{\Delta 100}$	5
pACAA1	pET28a expression plasmid containing C-terminally His_6 tagged *Cj*Pat$B_{\Delta 31}$	This study
pACAA2	pET28a expression plasmid containing C-terminally His_6 tagged *Cj*Pat$B_{\Delta 113}$	This study
pACAA3	pET28a expression plasmid containing C-terminally His_6 tagged *Cj*PatB	This study
pACAA4	pET28a expression plasmid containing N-terminally His_6 tagged *Cj*Pat$B_{\Delta 31}$	This study
pACAA5	pET28a expression plasmid containing N-terminally His_6 tagged *Cj*Pat$B_{\Delta 113}$	This study
pBS216[1]	pET-26b(+) derivative for expression of *Cj*PatA tandem fusion protein	This study
pJL002	pBS216 derivative for expression of (Y455F)*Cj*PatA	This study
pJL005	pBS216 derivative for expression of (H315A)*Cj*PatA	This study

Table S2: Strains, plasmids, and primers used in this study - *continued*

**Primers**		
*Cj*PatB$_{\Delta31}$ NdeI Fwd	CTAGCATATGCAAAAATATCACTTTGCATTCTATCC	IDT[2], this study
*Cj*PatB XhoI Rev	ATCGCTCGAGTTTACTAGCATTTTCTTCCTTAAGTTTT ATGTG	IDT, this study
*Cj*PatB FL CHis NcoI Fwd	ACGTCCATGGGCAGTGTAGTTAGATTTTTCTTTATTTT GATTATAGTG	IDT, this study
*Cj*PatB$_{\Delta113}$ NdeI Fwd	CTAGCATATGGATGCAAATATTAGTTTTATCGAC	IDT, this study
*Cj*PatB XhoI + stop Rev (N-His)	ATCGCTCGAGTTATTTACTAGCATTTTCTTCCTTAAGT TTTATGTG	IDT, this study
ABP16 Fwd	/5Phos/GGCACCGAATGGAAACAGGGC	IDT, this study
APB6r Rev	/5Phos/ACCACCAATCTGTTCTCTGTGAGCC	IDT, this study
oBS584	AATTTTGTTTAACTTTAAGAAGGAGATATACATATGAA ATACCTG	This study
oBS585	AGCCGGATCTCAGTGGTGG	This study
oJL003	CTCAAGCTTTTAATAACTCGAGCAC	This study
oJL004	AAAATAAAATCCGGAATGCCATCTG	This study
oJL008	GGGGAACACGCTGGCGTTTATTG	This study
oJL009	GCCCACATGCCTGACAGGATAAACG	This study

[1]pBS216 encodes SS$_{PelB}$-linker-His$_{10}$-linker-MBP-linker-[HRV 3C protease cleavage site]-linker *PatA from *C. jejuni* M1 (GenBank: ADN90800), where SS$_{PelB}$ = signal sequence of *Erwinia carotovora* pectate lyase B, MBP = *E. coli* maltose-binding protein. *Note: the *Campylobacter jejuni* PatA sequence was "codon-harmonized" for expression in *E. coli* using the Codon Harmonizer Galaxy server (6).
[2]IDT, Integrated DNA Technologies.

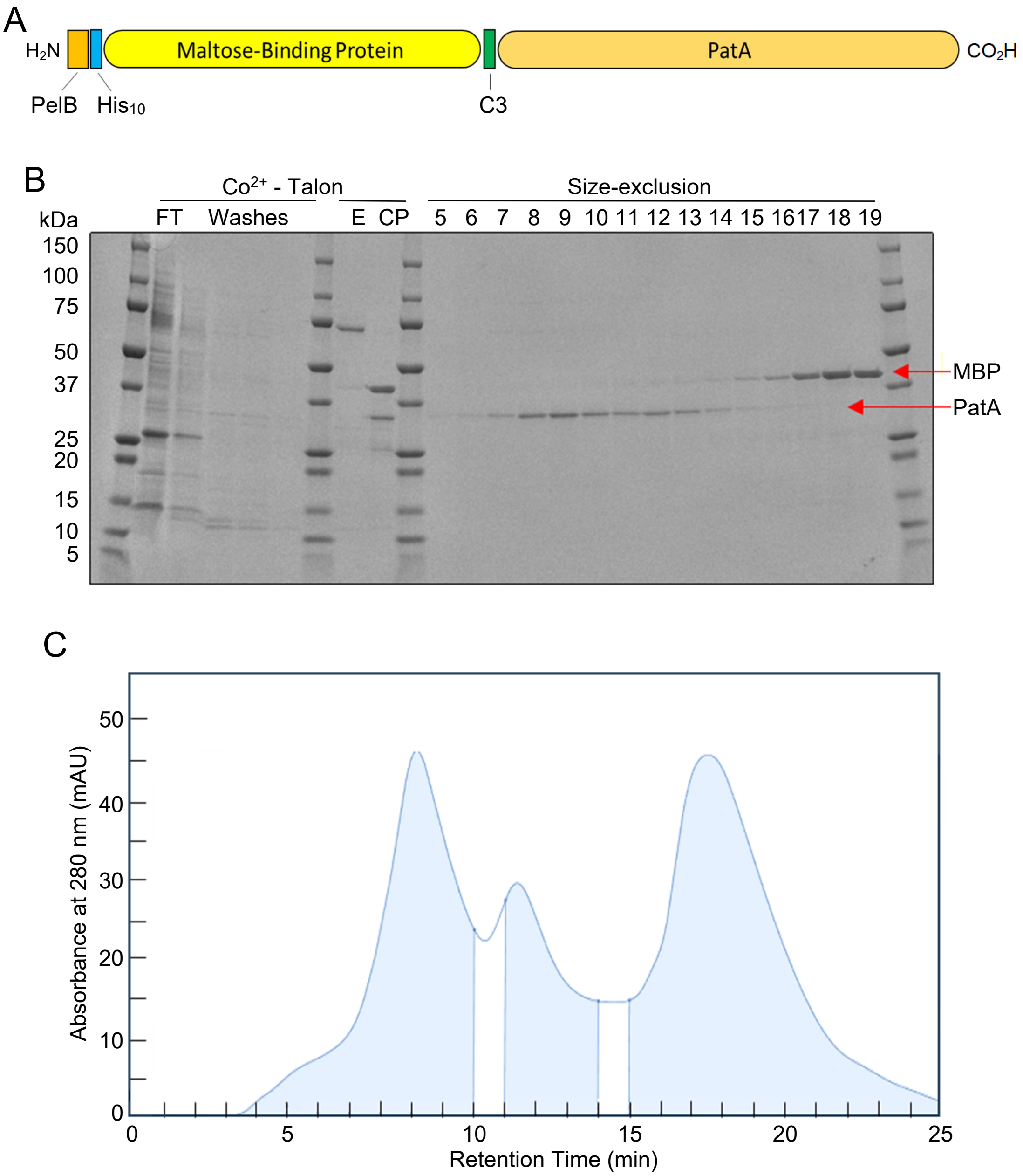

Figure S1. **Construct design and purification of recombinant PatA.** (A) The PatA expression construct design involving an N-terminal leader sequence (22 residues) from the pectate lyase gene *pelB* from *Erwinia caratovora*, followed by a deca-His tag, the *E. coli* maltose binding protein (MBP) lacking its N-terminal signal sequence, and a 3C protease recognition site (LEVLFQ/GP) fused in tandem to the 5' end of *patA*. (B) Representative SDS-PAGE analysis of PatA purification by Co^{2+}-Talon affinity and size-exclusion chromatographies. FT, column flow-through; E, column elution; CP, cleavage products of 3C protease. Lanes at right labelled 5 through 19 correspond to the respective numbered fractions of the chromatogram below (equal fraction volumes from the respective chromatographies were analyzed). (C) Representative chromatogram of fraction labelled CP in panel B subjected to size-exclusion chromatography. The blue shading identifies which fractions were pooled, respectively; purified PatA was recovered from pooled fractions 5–10, inclusive.

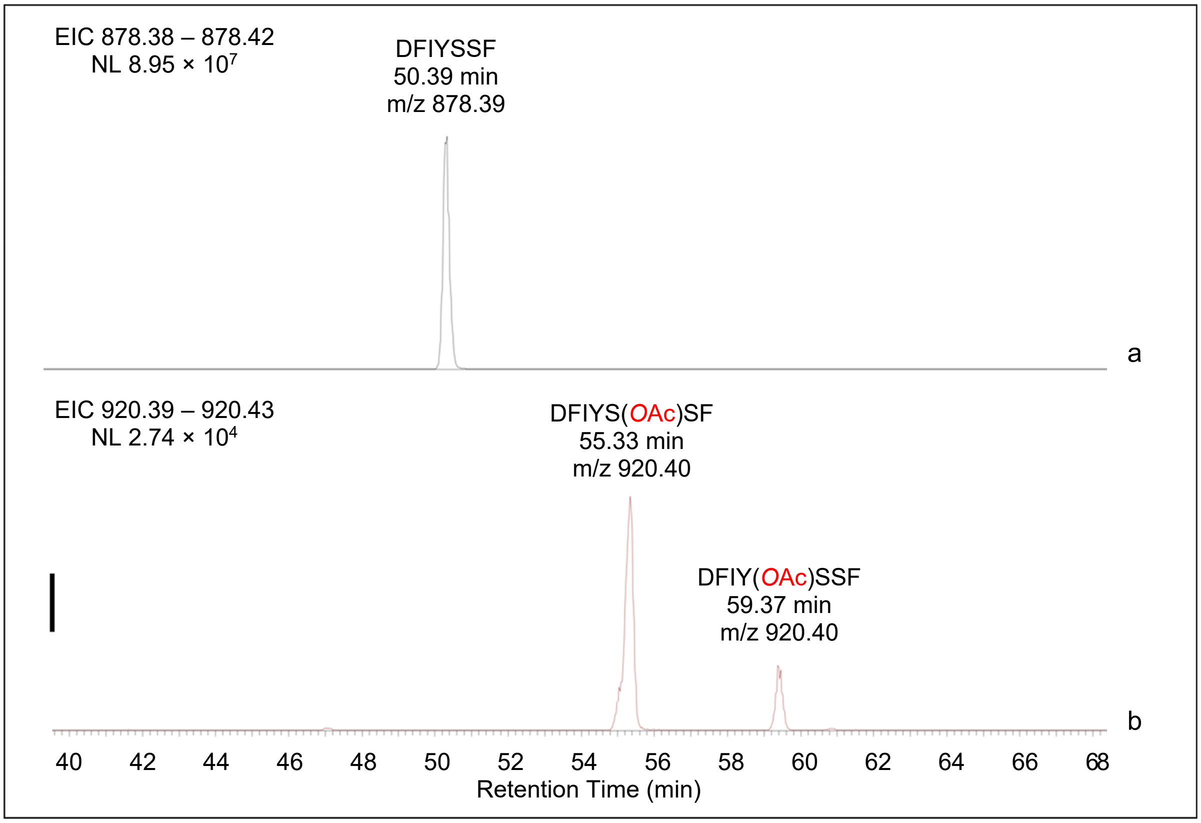

Figure S2. **Trapping of acetylated-PatA intermediate**. LC-MS analysis of AspN-digested PatA following its incubation with acetyl-CoA. Extracted ion chromatograms (EIC) of the (a) native and (b) acetylated DFIYSSF peptides. The solid bar on the left denotes 25% absorbance at 210 nm relative to largest peak.

Predicted Fragmentation Pattern

Seq	#	b: Δ Error	b	y	y: Δ Error	+1
D	1	---	116.034	---	---	7
F	2	-0.241	263.103	763.366	---	6
I	3	-0.137	376.187	616.298	-0.356	5
Y	4	-1.066	539.250	503.214	0.329	4
S	5	-6.546	626.282	340.150	0.051	3
S	6	---	713.314	253.118	0.069	2
F	7	---	---	166.086	0.291	1

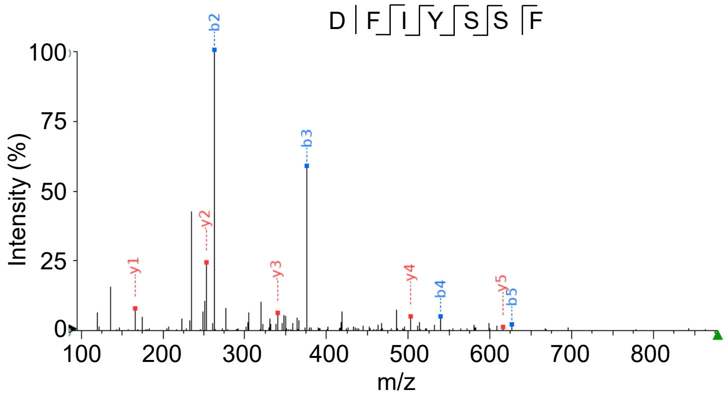

Predicted Fragmentation Pattern

Seq	#	b: Δ Error	b	y	y: Δ Error	+1
D	1	---	116.034	---	---	7
F	2	0.107	263.103	805.377	---	6
I	3	0.107	376.187	658.308	2.347	5
Y#	4	-1.314	581.261	545.224	0.853	4
S	5	3.995	668.293	340.150	-3.538	3
S	6	---	755.325	253.118	0.672	2
F	7	---	---	166.086	0.658	1

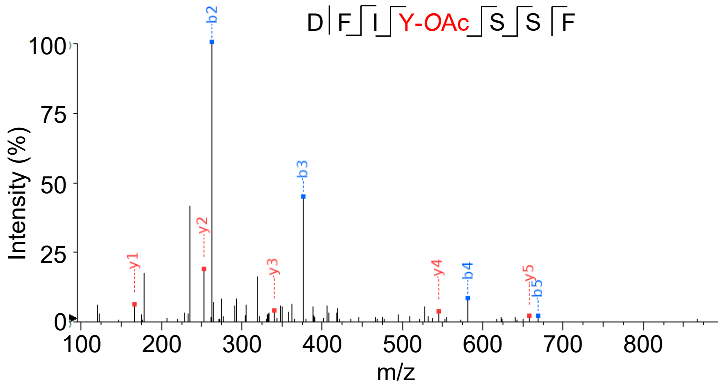

Predicted Fragmentation Pattern

Seq	#	b: Δ Error	b	y	y: Δ Error	+1
D	1	---	116.034	---	---	7
F	2	0.339	263.103	805.377	---	6
I	3	-0.056	376.187	658.308	---	5
Y	4	-0.953	539.250	545.224	1.189	4
S#	5	---	668.293	382.161	---	3
S	6	---	755.325	253.118	-0.172	2
F	7	---	---	166.086	1.577	1

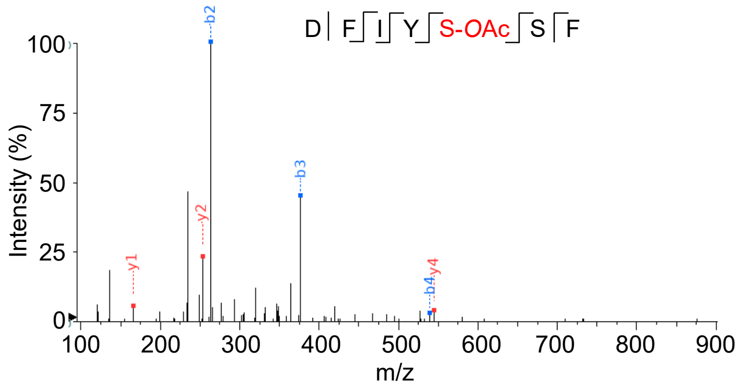

Figure S3. **Representative MS/MS analysis of the *O*-acetylated PatA peptides**. Fragment ion table (left) and MS/MS spectra (right) for the peptides as identified.

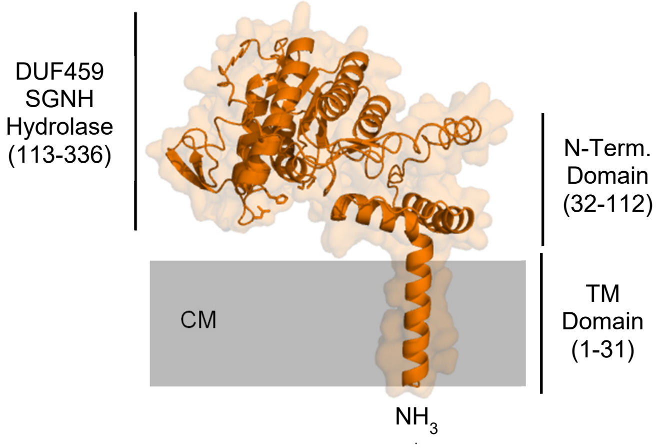

Fig. S4. Predicted structure of PatB. Cartoon presentation of an AlphaFold model of full-length *Cj*PatB topology. Numbering in parentheses denotes residues associated with the identified domains. CM, cytoplasmic membrane.

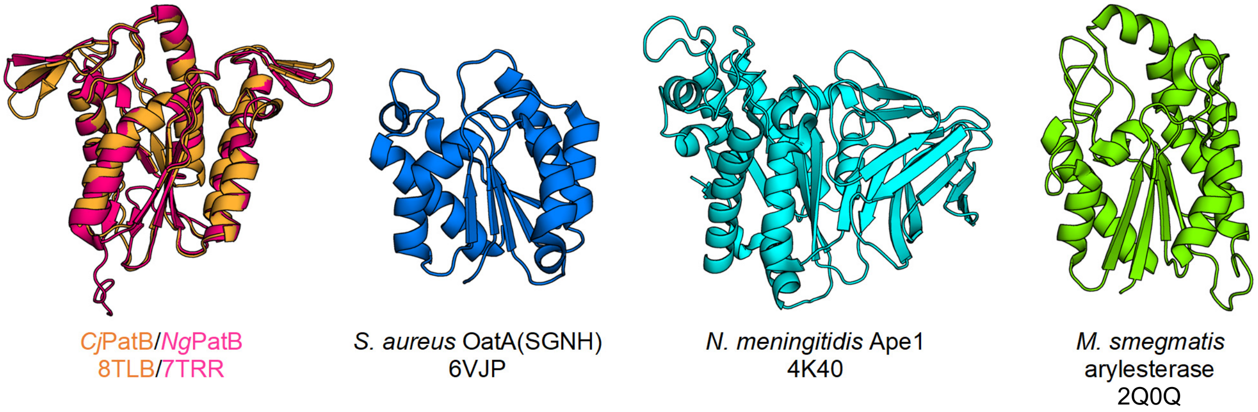

Figure S5. Comparison of the SGNH hydrolase domain of PatB with those of other family members. The superpositioned structures of PatB from *C. jejuni* (orange) and *N. gonorrhoeae* (magenta) contain the central α/β hydrolase fold characteristic of the SGNH hydrolases but in addition possess unique β-hairpin motifs not seen in other family members.

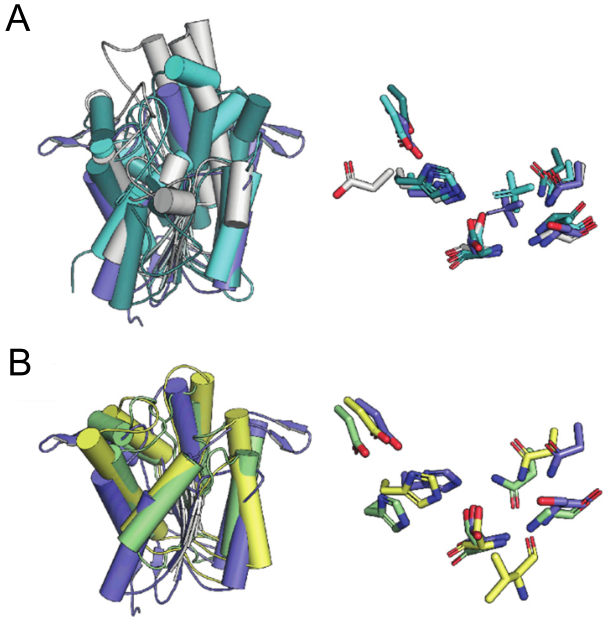

Figure S6. Structural comparison of *Ng*PatB$_{\Delta100}$ to proteins identified by the DALI server. Superposition of the full structures (left; cartoon form) and catalytic residues (right, stick form) of *Ng*PatB$_{\Delta100}$ (indigo) with (A) *M. smegmatis* arylesterase (cyan, PDB 2Q0Q), *S. albidoflavus* phospholipase (grey, PDB 4HYQ) and *G. stearothermophilus* acetylxylan esterase Axe2 (dark teal, PDB 3W7V), and (B) OatA_C from *S. aureus* (green, PDB 6VJP) and *S. pneumoniae* (yellow, PDB 5UFY).

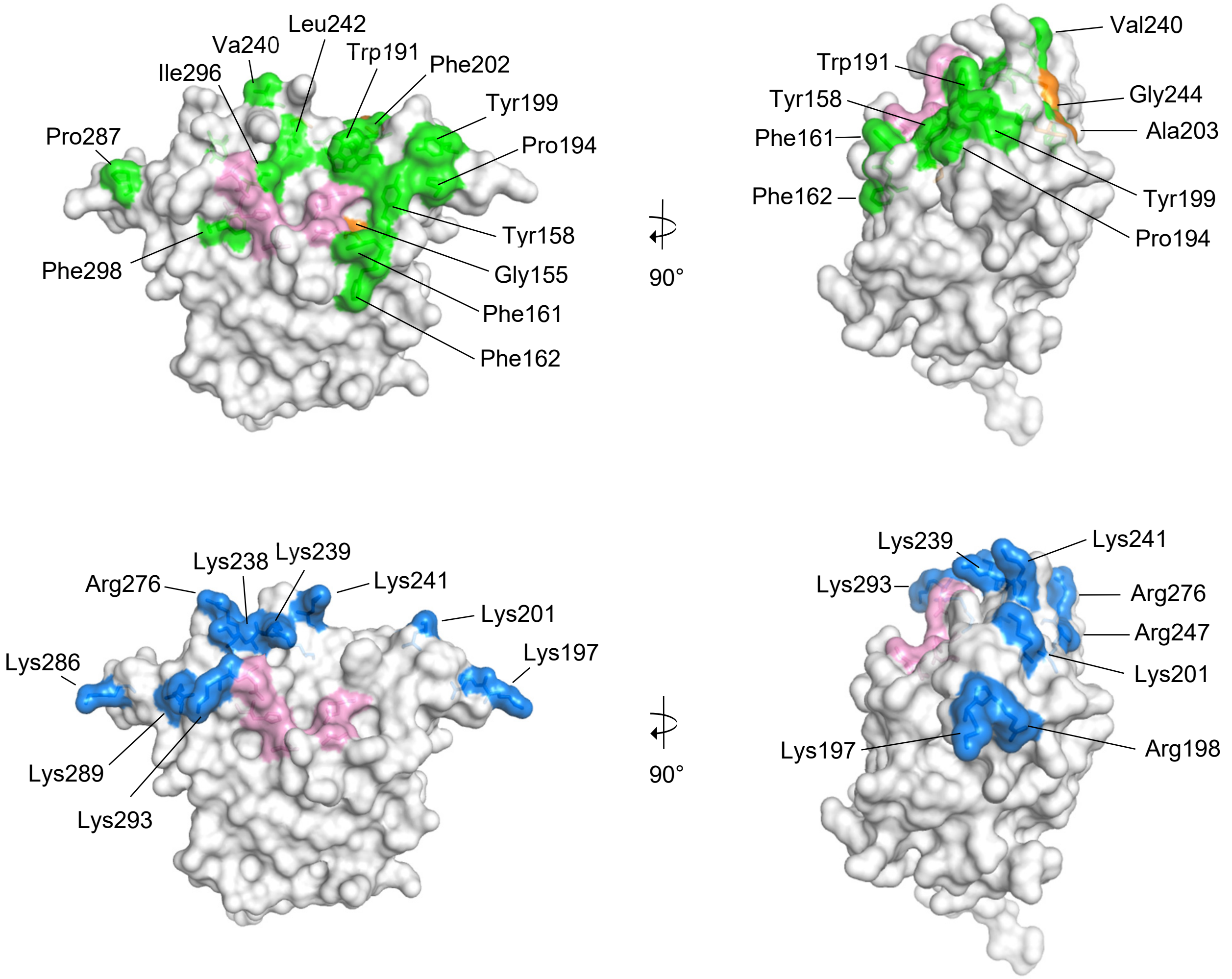

Figure S7. Surface non-polar and charged residues in *Ng*PatB. Surface-exposed non-polar (top) and positively-charge (bottom) residues in *Ng*PatB$_{\Delta 100}$ are depicted in green/orange and blue, respectively; the catalytic and oxyanion hole residues are in pink.

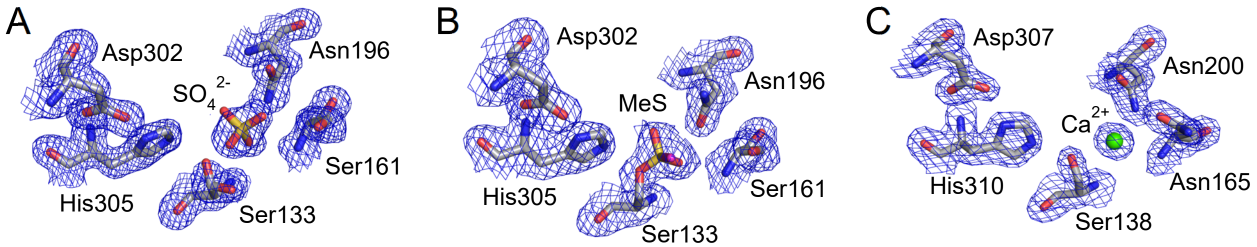

Figure S8. Accessory electron density in the PatB active sites. (A) The native structure of *Ng*PatB (7TJB) contains a tetrahedral electron density adjacent to Ser133 that can be attributed to a sulfate anion. Two discrete conformers of the Ser133 sidechain are observed oriented both toward and away from the His305 sidechain. (B) The MS-bound structure of *Ng*PatB (7TRR) contains a similar tetrahedral electron density, but this density is continuous with the Ser133 sidechain thus indicating a covalent MS adduct. Because there is no unique PDB ligand for *O*-methylsulfonyl-L-Ser, OSE (*O*-sulfo-L-Ser) was used for the depiction. (C) The native structure of *Cj*PatB (8TLB) displays only one conformer of the catalytic Ser138. A Ca^{2+} ion is observed occupying the same position as sulfate in the *Ng*PatB structure. All maps shown represent the 2Fo-Fc map contoured at 2σ.

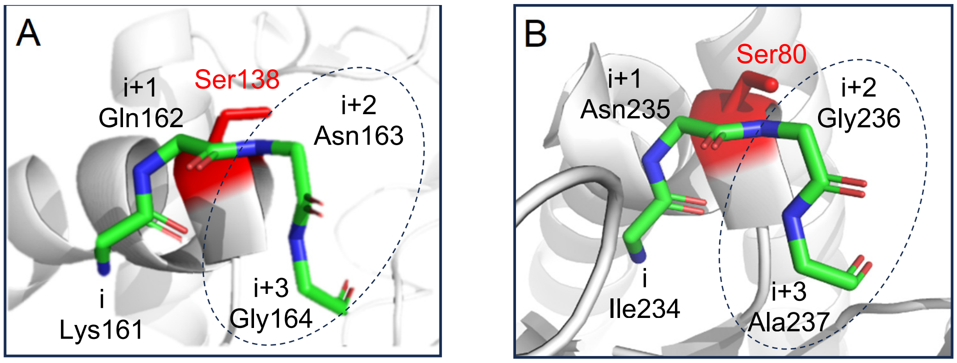

Figure S9. β-Turns of Block II residues in SGNH hydrolases. Stick presentation of the main chain atoms of the residues forming the (A) Type 1 and (B) Type 2 β-turns in *Ng*PatB and *N. mengitidis* Ape1 (PDB ID 4K40), respectively. The difference in the turns is seen with the orientation of the i+2 and i+3 residues (dashed circles). The respective catalytic Ser residues are in red.

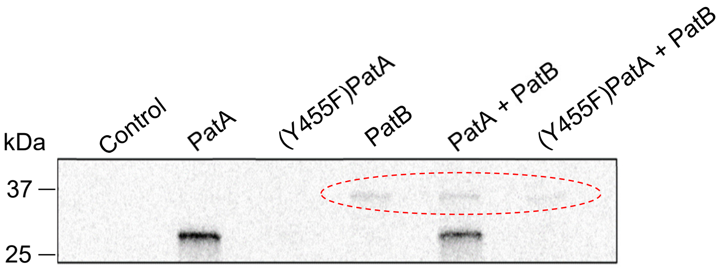

Figure S10. Detection of acetyl-transfer from PatA to PatB. The enzymes (5 µM) in 50 mM HEPES, pH 7.4 containing 150 mM NaCl were incubated with 100 µM [1,2-^{14}C]acetyl-CoA for 15 min prior to SDS-PAGE with autoradiography detection. The dashed red circle denotes the weak signal detected from PatB in both the absence and presence of PatA.

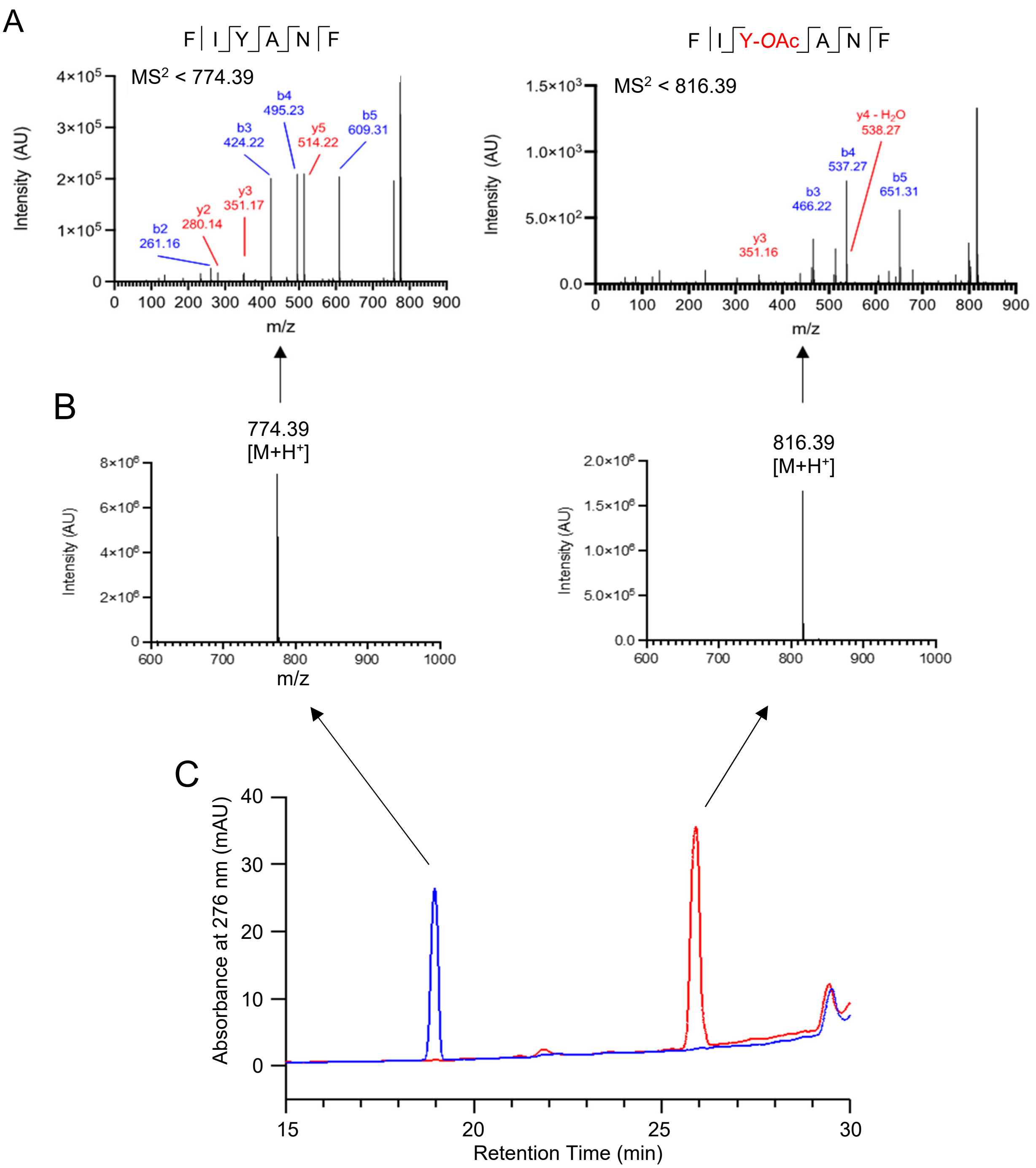

Figure S11. **Purification and assignment of the synthetic peptide product**. (A) Representative HPLC chromatogram tracings of the starting material (blue) and acetylated product (red) demonstrates stoichiometric acetylation of the peptide. (B) MS identification matches of the expected size of the starting material (773.4 Da) and confirms the product is singly acetylated (815.4 Da). (C) MS/MS fragmentation allows the assignment of the 42.0 Da acetyl group to the Tyr residue of the peptide.

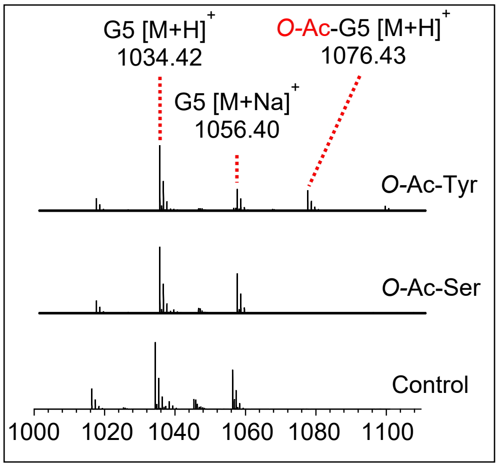

Figure S12. ***O*-Acetyl-tyrosine as a substrate for PatB.** Representative LC-MS analysis of reaction products of PatB acting as an *O*-acetyltransferase. *Cj*PatB (1 µM) in 50 mM sodium phosphate pH 7.0 was incubated with 5 mM chitopentaose as acceptor in the absence (control) or presence of 1 mM *O*-acetyl-Ser or *O*-acetyl-Tyr.

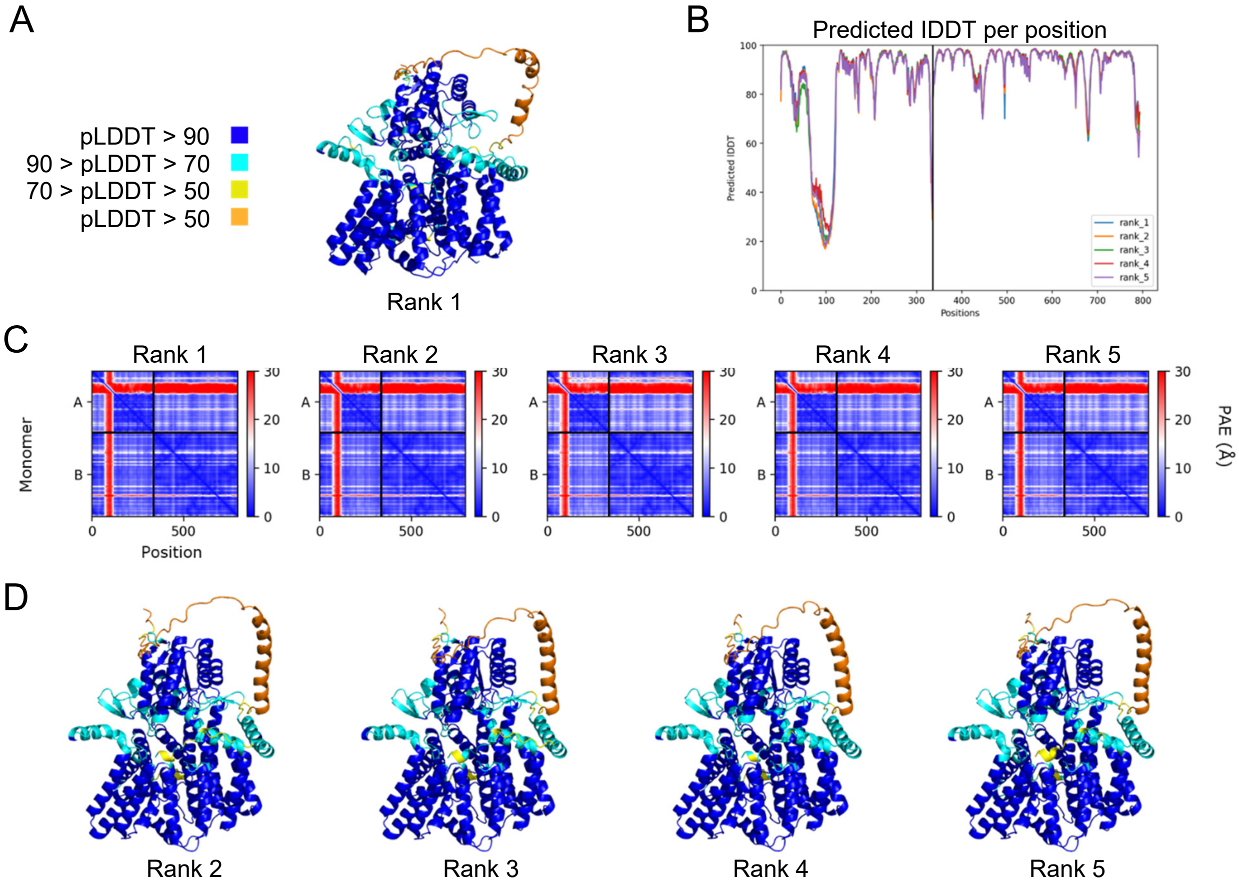

Figure S13. **AlphaFold Multimer model of *C. jejuni* PatAB**. (A) The top-scoring model of a PatAB complex. The model is coloured by predicted local distance difference test (pLDDT) values. Model confidence is based upon these scores which are quantized into very high (pLDDT > 90; blue), confident (90 > pLDDT > 70; cyan), low (70 > pLDDT > 50; yellow), and very low (50 > pLDDT; orange). (B) pLDDT scores as a function of residue for each of the top five scoring models. (C) The predicted-aligned error (PAE) heatmap for each of the top scoring models. Each x,y value represents the predicted coordinate error of residue x (in Å) between the true and predicted structures if they were aligned on position y. Monomer A is PatB and monomer B is PatA. (D) The 2nd to 5th ranked AlphaFold Multimer models of PatAB coloured by pLDDT as in panel A.

ATGAAATACCTGCTGCCGACCGCTGCTGCTGGTCTGCTGCTCCTCGCTGCCCAGCCGGCGATGG
CCGCAGATAGCGGTAGCTCTGGCCACCATCATCACCATCACCATCACCATCATGGCAGCTCTGG
TAGCAAAATCGAAGAAGGTAAACTGGTAATCTGGATTAACGGCGATAAAGGCTATAACGGTCTC
GCTGAAGTCGGTAAGAAATTCGAGAAAGATACCGGAATTAAAGTCACCGTTGAGCATCCGGATA
AACTGGAAGAGAAATTCCCACAGGTTGCGGCAACTGGCGATGGCCCTGACATTATCTTCTGGGC
ACACGACCGCTTTGGTGGCTACGCTCAATCTGGCCTGTTGGCTGAAATCACCCCGGACAAAGCG
TTCCAGGACAAGCTGTATCCGTTTACCTGGGATGCCGTACGTTACAACGGCAAGCTGATTGCTT
ACCCGATCGCTGTTGAAGCGTTATCGCTGATTTATAACAAAGATCTGCTGCCGAACCCGCCAAA
AACCTGGGAAGAGATCCCGGCGCTGGATAAAGAACTGAAAGCGAAAGGTAAGAGCGCGCTGATG
TTCAACCTGCAAGAACCGTACTTCACCTGGCCGCTGATTGCTGCTGACGGGGGTTATGCGTTCA
AGTATGAAAACGGCAAGTACGACATTAAAGACGTGGGCGTGGATAACGCTGGCGCGAAAGCGGG
TCTGACCTTCCTGGTTGACCTGATTAAAAACAAACACATGAATGCAGACACCGATTACTCCATC
GCAGAAGCTGCCTTTAATAAAGGCGAAACAGCGATGACCATCAACGGCCCGTGGGCATGGTCCA
ACATCGACACCAGCAAAGTGAATTATGGTGTAACGGTACTGCCGACCTTCAAGGGTCAACCATC
CAAACCGTTCGTTGGCGTGCTGAGCGCAGGTATTAACGCCGCCAGTCCGAACAAAGAGCTGGCA
AAAGAGTTCCTCGAAAACTATCTGCTGACTGATGAAGGTCTGGAAGCGGTTAATAAAGACAAAC
CGCTGGGTGCCGTAGCGCTGAAGTCTTACGAGGAAGAGTTGGCGAAAGATCCACGTATTGCCGC
CACTATGGAAAACGCCCAGAAAGGTGAAATCATGCCGAACATCCCGCAGATGTCCGCTTTCTGG
TATGCCGTGCGTACTGCGGTGATCAACGCCGCCAGCGGTCGTCAGACTGTCGATGAAGCCCTGA
AAGACGCGCAGACTGGCAGCGGCGGTAGCGGTGGCTCTCTGGAAGTGCTGTTTCAGGGCCCGAG
CGGTTCTGGTGGCAGCGGCAGCATTACCTATTTTTCGCTAGAATTTAGCATTCTGATGATCGCG
TTTTTTGCGATTTATTGGACCTTTAAAAACGATTATAAAATCCAGAACATTCTGATTCTGATTT
TCTCGTATATTATTTATATTCTGATAAACCCGTATTTCGCTCTGGTGCTGTTTATCTATACATT
TTTTATCCACTATTTCGCTTTACTAATTTTTGTACGGCGGAAGCGGTATATTTTCGCGACCTGT
ATGGCGTTTATTATTCTGAACCTGTGCTTTTTTAAATACTTTCCGTCCATAAAAGGCAGCGTGG
ATGAGATCCTGAACTTTTTCGGCCTGGAATTTCTGAACATTGATCTGGTTCTGCCAATCGGCAT
TAGCTTTTATACATTTACCTCGATAACCTATCTGGTAGAAGTGTATCAGAAACGGCGGCTGGAA
AGCTTTCTGAACCTGGCAACATTCCTGTCATTTTTTCCAACGCTACTGTCAGGACCGATTATGA
GGAGCTCGTTTTTCTTTGAACAAGCGTATCAGAAACGGGAATTTAAACATGCAAACCTGATAAT
AATATTACTGGTGTTTGGCATCGTGAAAAAAGTGCTGATCGCTAACTACCTGGGCATTTATGCG
AAAAGCATTCTGGACTTTCCACAGTCGTATAACTTCATCCAGCTGCTGTCCGCAATTTACGCTT
ATGCAATTCAGATCTACTGTGATTTTTCCGGCTATGTGGATCTGGTGTGCGCGTTTGCGCTGAT
GCTGGGCTTTACACTGCCCCCGAATTTTAATATGCCGTATCTGGCAAAAAATCTAAAAGATTTT
TGGGCGCGCTGGCATATAAGCCTGTCAACATTTATCCGCGATTATATATATATCCCGCTGGGAG
GGAACCGCAAAGGAATCCCACGGACCGTTGCGAACATCCTAATAGCGTTTATCCTGTCAGGCAT
GTGGCATGGGAACACGCTGGCGTTTATTGTGTGGGGCCTGTTACATGGCATTGGGATCGTGTTT
ATCCATTTACTAACCCTGTCCAAATTTAGCCTGCAGAAAATTCCAGCGCTGGGCCGCTTTCTGA
CATTTCAGTTTGTTTGCTTTACCTGGATTTTCTTTTATTATTCCAAAAACCTAGAAGATGCAAT
CGAATATTTTAAAGCGTGCTATTATAACTTCTTCCAGATTCCATCGTATAATGATATATATATG
TTAGTGGCGTTTGGAGTGCTGTTTATGATATATCCGCTGTTTATTAACTTTAAAGAATATTGTA
TTAAGATTCTGAACCTGACCCCATTTCTGCTAAAACCGTTTATAATCGCGTTTATTCTGCTGTT
AGTGTTTGCGTTTATGCCAGATGGCATTCCGGATTTTATTTATTCAAGCTTTTAATAA

Figure S14. DNA coding sequence for PelB-His$_{10}$-MBP-3C-*Cj*PatA expression construct.

MKYLLPTAAAGLLLLAAQPAMAADSGSSGHHHHHHHHHHGSSGSKIEEGKLVIWINGD
KGYNGLAEVGKKFEKDTGIKVTVEHPDKLEEKFPQVAATGDGPDIIFWAHDRFGGYAQ
SGLLAEITPDKAFQDKLYPFTWDAVRYNGKLIAYPIAVEALSLIYNKDLLPNPPKTWEEIP
ALDKELKAKGKSALMFNLQEPYFTWPLIAADGGYAFKYENGKYDIKDVGVDNAGAKA
GLTFLVDLIKNKHMNADTDYSIAEAAFNKGETAMTINGPWAWSNIDTSKVNYGVTVLP
TFKGQPSKPFVGVLSAGINAASPNKELAKEFLENYLLTDEGLEAVNKDKPLGAVALKSY
EEELAKDPRIAATMENAQKGEIMPNIPQMSAFWYAVRTAVINAASGRQTVDEALKDAQ
TGSGGSGGSLEVLFQGPSGSGGSGSITYFSLEFSILMIAFFAIYWTFKNDYKIQNILILIFSYI
IYILINPYFALVLFIYTFFIHYFALLIFVRRKRYIFATCMAFIILNLCFFKYFPSIKGSVDEILN
FFGLEFLNIDLVLPIGISFYTFTSITYLVEVYQKRRLESFLNLATFLSFFPTLLSGPIMRSSFF
FEQAYQKREFKHANLIIILLVFGIVKKVLIANYLGIYAKSILDFPQSYNFIQLLSAIYAYAIQ
IYCDFSGYVDLVCAFALMLGFTLPPNFNMPYLAKNLKDFWARWHISLSTFIRDYIYIPLG
GNRKGIPRTVANILIAFILSGMWHGNTLAFIVWGLLHGIGIVFIHLLTLSKFSLQKIPALGR
FLTFQFVCFTWIFFYYSKNLEDAIEYFKACYYNFFQIPSYNDIYMLVAFGVLFMIYPLFIN
FKEYCIKILNLTPFLLKPFIIAFILLLVFAFMPDGIPDFIYSSF**

Figure S15. Amino acid sequence for PelB-His$_{10}$-MBP-3C-*Cj*PatA expression construct.

SI References

1. Chen, V. B., Arendall, W. B., Headd, J. J., Keedy, D. A., Immormino, R. M. Immormino, Kapral, G.J. *et al.* (2010) MolProbity: All-atom structure validation for macromolecular crystallography. *Acta Crystallogr. Sect. D Biol. Crystallogr.* **66**, 12–21.
2. Korlath, J.A., Osterholm, M.T., Judy, L.A., Forfang, J.C., and Robinson, R.A. (1985) A point-source outbreak of campylobacteriosis associated with consumption of raw milk. *J. Infect.Dis*. **152**, 592–596 .
3. Miroux, B., and Walker, J.E. (1996) Over-production of proteins in *Escherichia coli*: Mutant hosts that allow synthesis of some membrane proteins and globular proteins at high levels. *J. Mol. Biol.* **280**, 289–298 .
4. Dempsey, J.A., Litaker, W., Madhure, A., Snodgrass, T.L., and Cannon, J.G. (1991) Physical map of the chromosome of *Neisseria gonorrhoeae* FA1090 with locations of genetic markers, including opa and pil genes. *J. Bacteriol.* **173**, 5476-5486.
5. Brott, A.S., Jones, C.S., and Clarke, A.J. (2019) Development of a high throughput screen for the identification of inhibitors of peptidoglycan *O*-acetyltransferases, new potential antibacterial targets. *Antibiotics*. **8**, 65.
6. https://galaxyproject.org/use/codon-harmonizer/
